# What is the best fitness measure in wild populations? A case study on the power of short-term fitness proxies to predict reproductive value

**DOI:** 10.1371/journal.pone.0260905

**Published:** 2022-04-22

**Authors:** Živa Alif, Jamie Dunning, Heung Ying Janet Chik, Terry Burke, Julia Schroeder

**Affiliations:** 1 Department of Life Sciences, Imperial College London, Silwood Park, Ascot, Berkshire, United Kingdom; 2 Groningen Institute for Evolutionary Life Sciences, University of Groningen, Groningen, The Netherlands; 3 Department of Animal and Plant Sciences, University of Sheffield, Sheffield, United Kingdom; University of Tulsa, UNITED STATES

## Abstract

Fitness is at the core of evolutionary theory, but it is difficult to measure accurately. One way to measure long-term fitness is by calculating the individual’s reproductive value, which represents the expected number of allele copies an individual passes on to distant future generations. However, this metric of fitness is scarcely used because the estimation of individual’s reproductive value requires long-term pedigree data, which is rarely available in wild populations where following individuals from birth to death is often impossible. Wild study systems therefore use short-term fitness metrics as proxies, such as the number of offspring produced. This study compared two frequently used short-term metrics for fitness obtained at different offspring life stages (eggs, hatchlings, fledglings and recruits), and compared their ability to predict reproductive values derived from the genetic pedigree of a wild passerine bird population. We used twenty years of precise field observations and a near-complete genetic pedigree to calculate reproductive success, individual growth rate and de-lifed fitness as lifetime fitness measures, and as annual de-lifed fitness. We compared the power of these metrics to predict reproductive values and lineage survival to the end of the study period. The three short-term fitness proxies predict the reproductive values and lineage survival only when measured at the recruit stage. There were no significant differences between the different fitness proxies at the same offspring stages in predicting the reproductive values and lineage survival. Annual fitness at one year old predicted reproductive values equally well as lifetime de-lifed fitness. However, none of the short-term fitness proxies were strongly associated with the reproductive values. The commonly used short-term fitness proxies best predict long-term fitness when measured at recruitment stage. Thus, because lifetime fitness measured at recruit stage and annual fitness in the first year of life were the best proxies of long-term fitness in short-lived birds, we encourage their future use.

## Introduction

The concept of fitness is central to evolutionary theory [1]. Natural selection maximises fitness, which is therefore a driving force of evolution as well as a measure of evolutionary success [2]. One definition of relative fitness is how good an individual is at spreading its genes into future generations, relative to all other individuals in the population [2]. A universal definition of fitness in mathematical terms that applies to all population structures and dynamics is however not agreed on [2–5].

Ecological studies measure fitness in diverse ways, often depending on the research question, the population dynamics, and the ecology of the study species [6, 7]. While some studies measure fitness across lifetimes, others measure individual annual fitness to examine variation in selection between years [8]. Lifetime fitness measures are considered more accurate than annual measures, as the latter is influenced by environmental stochasticity [7, 9]. Alternative fitness metrics have been developed that account for environmental stochasticity and population dynamics [5, 10–12].

Studies use different metrics of fitness—some fitness metrics include both survival and fecundity components [8, 13], while others focus on only one component as a proxy, such as lifespan [4], or on only a single life-history trait, such as the age at first reproduction [13, 14]. Two frequently used individual fitness metrics are lifetime reproductive success (LRS) [15] and individual growth rate (IGR) [13]. Both count the number of offspring produced in the individual’s lifetime, but IGR gives more weight to offspring produced at a younger age [13], therefore results differ [16]. Other individual fitness metrics can also include survival–for example de-lifed fitness is composed of individual’s annual survival and fecundity relative to the rest of the population. Different fitness proxies do not necessarily correlate with each other well [7] and more research is needed to determine which is the most appropriate measure of fitness [6]. Population dynamics also affects the accuracy of different fitness measures–for example, IGR has been developed specifically for growing populations and is the best fitness measure for such cases, while LRS is more accurate in stable or declining populations [6]. Choosing the appropriate fitness metric is therefore an important consideration when designing a study [7].

When measuring fitness it is also important to consider age of individual’s offspring at the measurement time, Fitness should ideally be measured zygote-to-zygote because at older offspring ages, offspring survival can be seen as a part of offspring fitness due to its unique genotype that affects survival probability, or a part of parental fitness in cases where parental phenotype affects offspring mortality, for example through parental care [17]. Counting offspring at higher stages of development assigns a part of offspring fitness into parental fitness, thus potentially affecting the strength and direction of selection. Furthermore, Brommer et al. [6] show that the age of offspring census directly affects IGR values, while it does not necessarily affect LRS, making the two fitness proxies less comparable at different stages. Thus, it is important to understand how census time affects the measure of parental fitness, particularly given that measuring fitness zygote-to-zygote is challenging in practice and studies count offspring at varying ages or life-history stages.

Although fitness is considered to be a measure of an individual’s gene copy frequency in future generations, most fitness metrics are only short-term and focus solely on an individual’s direct descendants. Alternatively, the reproductive value from a single individual, defined as the expected number of copies of each of an individual’s alleles in a future generation conditional on a realised pedigree of descendants, can be used to measure long-term fitness [18]. The reproductive values can be estimated from a genetic pedigree, following rules of mendelian inheritance to calculate how many allele copies survive on average. The reproductive values stabilise after log_2_*N* generations, where *N* is the population size, which corresponds to about 7 generations for a population of 100 and only 13 generations for a population of 10,000. [4, 18, 19]. The reproductive values are therefore determined in ~10 generations and closely correspond to the long-term probability of the persistence of a specific allele in a population. As such, reproductive value predict individual’s ultimate genetic contribution that only emerges over long timescales (>100 generations). [18].

While reproductive values closely predict allele survival probability, the realized genetic contributions in terms of allele frequencies usually differ greatly [18]. Due to recombination and segregation in meiosis the actual genetic frequencies, conditional on allele survival, follow a random distribution [18–21], and not all genealogical ancestors are necessarily also genetic ancestors [22]. Although the actual ultimate genetic frequencies cannot be estimated from the pedigree due to the randomness in recombination and meiosis, reproductive value is closely associated with allele survival probability, which is an indicator of individual’s success of passing its genes to future generations. As such, reproductive value is a practical and relevant measure for evolutionary studies and is maximised by natural selection, thus closely corresponding to fitness [18, 23].

This study examined the correlation between several short-term fitness metrics (IGR, LRS and de-lifed fitness), and reproductive values. We examined three commonly used individual short-term fitness metrics [24]: lifetime reproductive success (LRS) and individual growth rate (IGR), which are based on fecundity only [13], and de-lifed fitness which includes fecundity and survival adjusted for population growth [8]. We measured the first two at four different developmental stages of offspring (eggs, hatchlings, fledglings, and recruits) to investigate at which offspring developmental stage fitness measures are most accurate. Unlike LRS and IGR, de-lifed fitness is a relative fitness measure which can be used to estimate both annual and lifetime fitness, and can be estimated only for recruit stage. Therefore, de-lifed fitness was compared to IGR and LRS only at recruit stage, but was also used as an annual fitness metric at different parent ages. We used data from an isolated native house sparrow (*Passer domesticus*) population on Lundy Island (United Kingdom) with 20 years of life history data, including near-complete genetic pedigree [25], precise measures of both survival, given the 0.96 yearly resighting rate [26], and reproductive success, as 95% of birds breed in monitored nest boxes [27].

## Methods

### Study system

**Fig 1:**
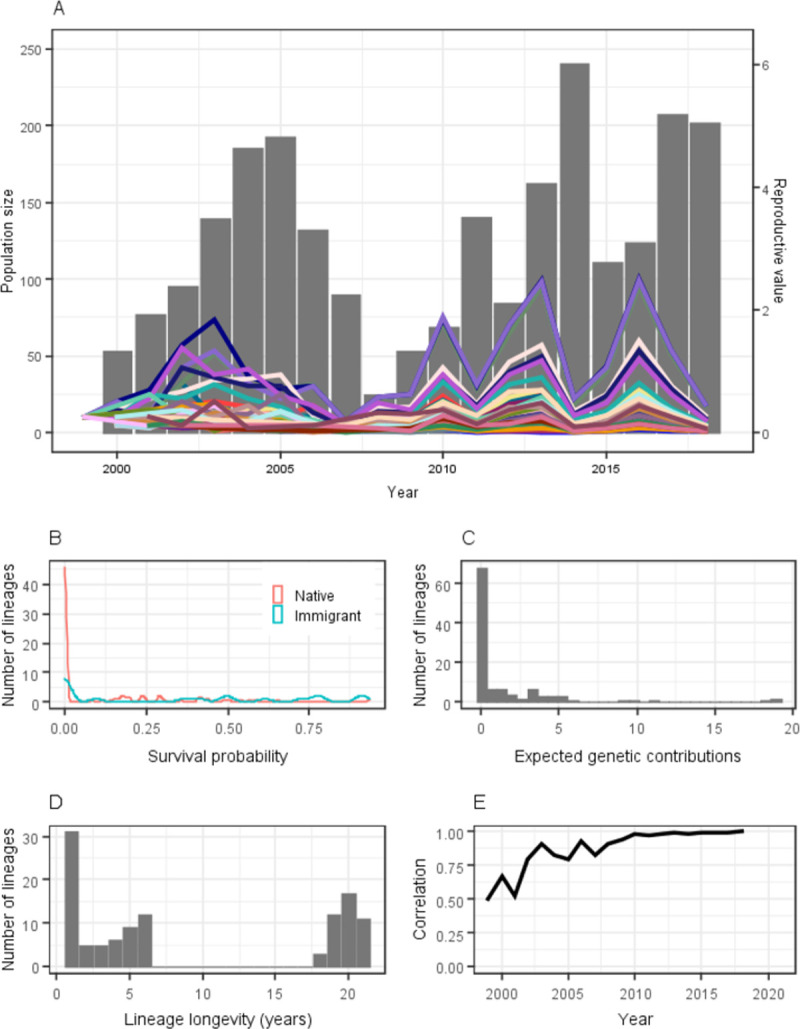
(A) Reproductive values and population size during the study period. Bars represent the population size and lines represent the reproductive values of each of the 43 lineages that survived to 2018. (B) Survival probability of 111 house sparrow lineages on Lundy Island from 1999 to 2018. (C) Reproductive values of the 111 lineages. (D) Number of years to lineage extinction for the 111 lineages. Lineages that survived 18, 19, 20 or 21 years are those that were still extant in 2018, corresponding to cohorts 1999, 2000, 2001 and 2002 respectively. (E) Correlation between the reproductive values in each year and the final year.

The native house sparrow population on Lundy Island has been continuously monitored since 2000. Lundy is a small island 19 km off the south-west English coast (51°11N, 4°40W). In addition to the native population, 50 individuals were brought to Lundy from the mainland in 2000 for an experiment [28]. Due to the distance of the island from the mainland and the sedentary nature of sparrows, there is minimal dispersal to and from the island [25]. The adult sparrow population size including both native and introduced birds has fluctuated between 25 and 241 individuals between 1999 and 2019 (Fig 1A), with the geometric mean population size of 110.9 individuals and the arithmetic mean population size of adult individuals of 126.3. The average lifespan of recruits was 3.0 years and the generation time, calculated as the mean parent age, was 2.35 years. The mean pedigree depth was 11.7 with a maximum of 17 and a minimum of 6 generations. Based on the theoretical predictions of reproductive values stabilising in *Glog*_2_(*N*) years [19], where *G* is the number of generations, the stabilisation in our study should occur in 16. 4 years or sooner as this estimate is based on arithmetic population size rather than normally smaller effective population size.

During systematic annual monitoring, each sparrow is ringed with three colour rings and one metal ring from the British Trust for Ornithology. Since most sparrows are initially caught as nestlings and ringed as fledglings, we know the identities of the parents attending their nests, and the exact age of almost all individuals [29]. Over 99% of the population has been ringed since 2000 [29]. If an individual is not seen for two years or more, it is assumed dead, with this assumption based on previous mark–recapture success data [26, 29]. Blood samples are collected upon bird capture and genotyped at up to 23 microsatellite loci [25]. This allows for the assignment of genetic parentage with 95% confidence [25]. From the genetic pedigree and the social brood information, the reproductive success of individuals is calculated. Thanks to these data, the study system provides unusually accurate survival, reproduction, and pedigree data for the complete population [25].

### Pedigree analysis

We calculated fitness proxies and the reproductive values for founders and half-founders that were born between 1999 and 2002. Founders and half-founders were defined as individuals for which both parents, or one parent, respectively, were unknown. To calculate reproductive values, we used our genetic pedigree containing all reproducing individuals up to 2018. While we initially calculated reproductive value for all founders, those from 2003 onwards were removed from subsequent analyses as they could not have been real population founders.

Reproductive values were calculated using gene dropping [30]. Gene dropping is a computer simulation in which each individual is assigned two alleles (one paternal and one maternal), and their Mendelian transmission down the pedigree is simulated. By repeating this simulation many times and calculating the mean values, robust estimates of reproductive values can be obtained by examining the frequency of an individual’s alleles in subsequent generations. In addition, the allele survival probability can be calculated by examining in how many simulations the allele survives in present-day individuals. We ran the simulation 10,000 times using R package nadiv [31]. Using the results, we derived lineage longevity, reproductive values, and allele survival probability. We define lineage longevity as the number of years before a lineage originating from one individual goes extinct, and allele survival probability is the proportion of gene dropping simulations in which a lineage survives. We explored whether lineages from the experimentally introduced sparrows differed from native lineages in their rate of survival to 2018 (last year with complete data) and in their reproductive values. We chose to work with years rather than generations as a measure of time because sparrows have overlapping generations.

### Short-term fitness metrics

We calculated the short-term fitness proxies for the founders and half-founders from cohorts between 1999 and 2002 with complete life-history data. Founders with incomplete life-history data were removed as these were mainly birds that were born prior to the beginning of data collection, hence could lead to an underestimation of their reproductive success. The individual lifetime production of eggs, broods, hatchlings, fledglings, and recruits was then calculated (LRS), as well as IGR at all four offspring developmental stages, and de-lifed fitness. Hatchlings were defined as offspring counted in a nest two days after hatching, and fledglings were birds that survived until ringing, which is typically 12 days after hatching. Recruits were defined as offspring that successfully reproduced and produced at least one egg in any subsequent years.

The IGR is the dominant eigenvalue of an individual population transition matrix, as described in [13]. In an individual population transition matrix, the sub-diagonal represents survival, and the first row is filled with the number of offspring produced at each parental age, divided by two because each parent only contributes half the alleles of its offspring. An example of an individual population transition matrix for an individual that survived three years and had 1, 2 and 1 offspring at ages 1, 2, and 3 respectively, is given below:

$$\left[\begin{array}{ccc} 0.5 & 1 & 0.5\\ 1 & 0 & 0\\ 0 & 1 & 0 \end{array}\right]$$

We also calculated annual de-lifed fitness based on the formula [8]:

$$p_{ti}=\frac{\xi_{t(i)}-w_{t}}{N_{t}-1}$$

Where:

- *p_ti_* is individual fitness
- *ξ*_*t*(*i*)_ is the number of individual’s surviving offspring at the end of the time step plus one if the individual survived
- *w_t_* is the population size in year *t*+1 divided by population size in year *t*
- *N_t_* is the population size of adults on 1^st^ April each year

While LRS and IGR are both lifetime fitness measures, de-lifing was designed primarily as a per-generation fitness proxy and is here calculated annually. However, lifetime de-lifed fitness can be obtained by summing the annual fitness values for each individual [8, 32]. We therefore used de-lifed fitness as both an annual fitness proxy and, after summing, as a lifetime fitness proxy.

We calculated Pearson’s correlation between each fitness metric and the reproductive values for the lineages that survived. We ran a binary logistic regression model in R version 4.0.3 [33], using MCMCglmm [34] with lineage survival to 2018 as the response variable, and each fitness metric as the explanatory variable. The fitness metrics were z-transformed so that the slopes were not affected by the variable variances. We used priors with the residual variance fixed at 0.5 and ran the model for 100,000 iterations with the thinning interval set at 70 and the burn-in at 7,000. We examined which fitness metric had the strongest association with lineage survival based on the slope of the regression.

### Ethics statement

As this was a theoretical study using previously selected data, no ethics approval was required.

## Results

### Reproductive values

There were in total 111 lineages arising between 1999 and 2002 used for the analyses. Of these 111 lineages, 18 lineages were founded by sparrows experimentally introduced in 2000 [28] and 93 lineages from native sparrows already present on the island in 2000. 43 lineages survived to 2018, of which 11 were introduced and 32 were native. Hence, at most, 39% of the founders passed genetic material to 2018, and there was only weak evidence of an association between a lineage’s origin and survival (*p* = 0.06, Fisher’s exact test). The mean allele survival probability was 0.16 (95% CI 0.13–0.18), and the survival probability for lineages appearing in 2018 was 0.40 (95% CI 0.37–0.43, Fig 1B). There was variation in the absolute reproductive values (mean = 1.64, 95% CI 1.32–1.95 range: 0.41–18.89, Fig 1C). The introduced lineages had on average higher reproductive values (4.99) than native lineages (0.99, W = 1117.5, p = 0.002)). Contributions varied over time, but after 2007 fluctuations were more synchronous among lineages, and the ranking of lineages based on their reproductive values remained similar (Fig 1A). Population fluctuations closely follow fluctuations in the reproductive values in the previous year. The change in lineage behaviour after 2007 is visible in lineage longevity, as all lineages that survived the from 2000 to 2006 also survived until 2018 (Fig 1A). After 2007 the correlation between annual reproductive value and reproductive values in 2018 also increased, and stabilised around 2011 subsequent to which the correlation was above 0.95 (Fig 1E).

### Fitness proxies

Fitness proxies were calculated for 86 founders, 44 males and 42 females. We estimated the correlation with the reproductive values of 42 lineages that survived to 2018 and had no missing fitness data. In total, individuals included in the analysis produced 2,054 eggs of which 1,746 (85%) survived to hatching, 881 (43%) to fledging and 294 (14%) recruited into the breeding population.

**Fig 2:**
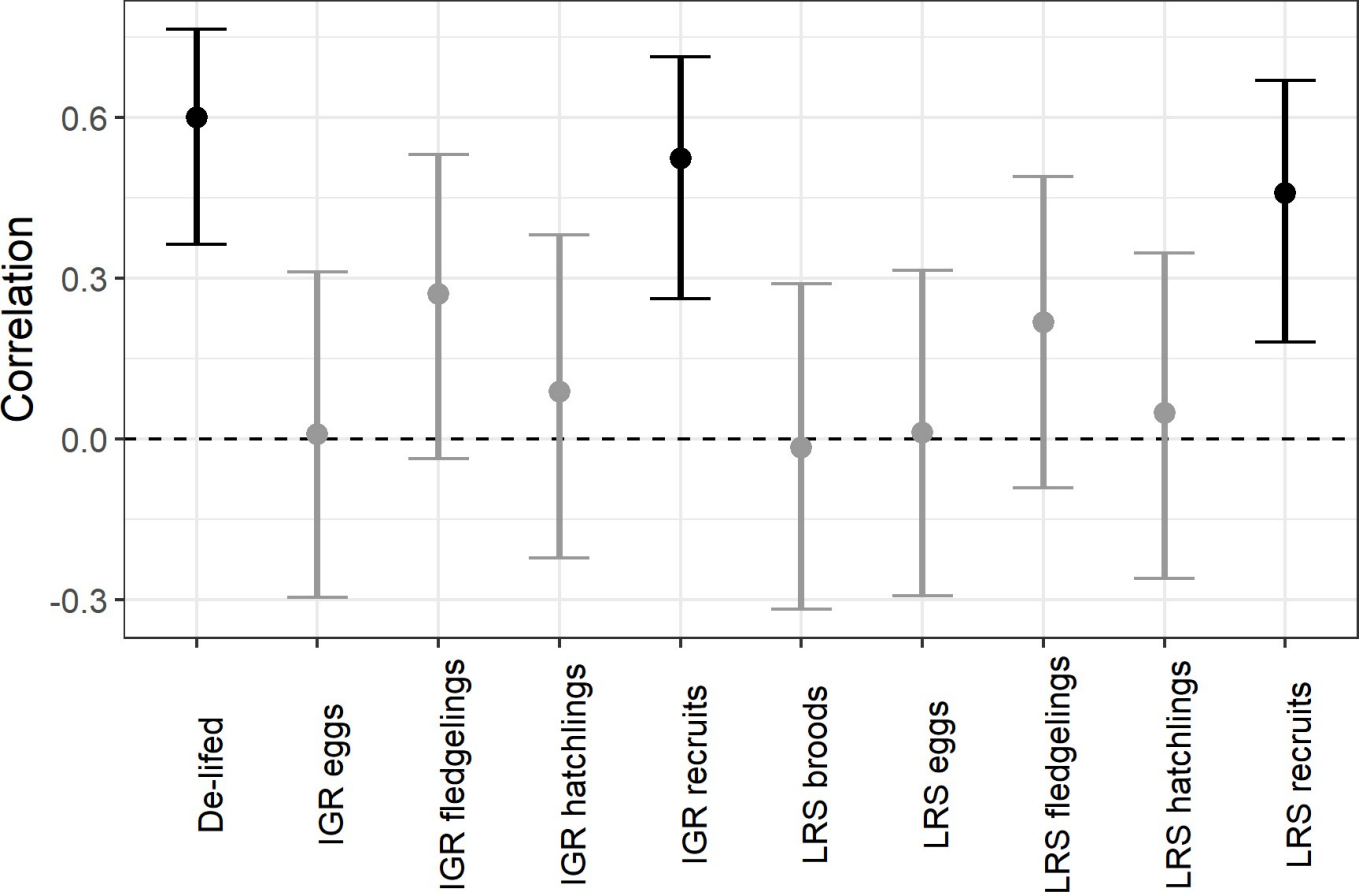
Correlation between each of the fitness proxies at different life stages with reproductive value. Error bars represent 95% confidence intervals. Black bars represent significant results, while light grey bars represent non-significant results.

**Fig 3:**
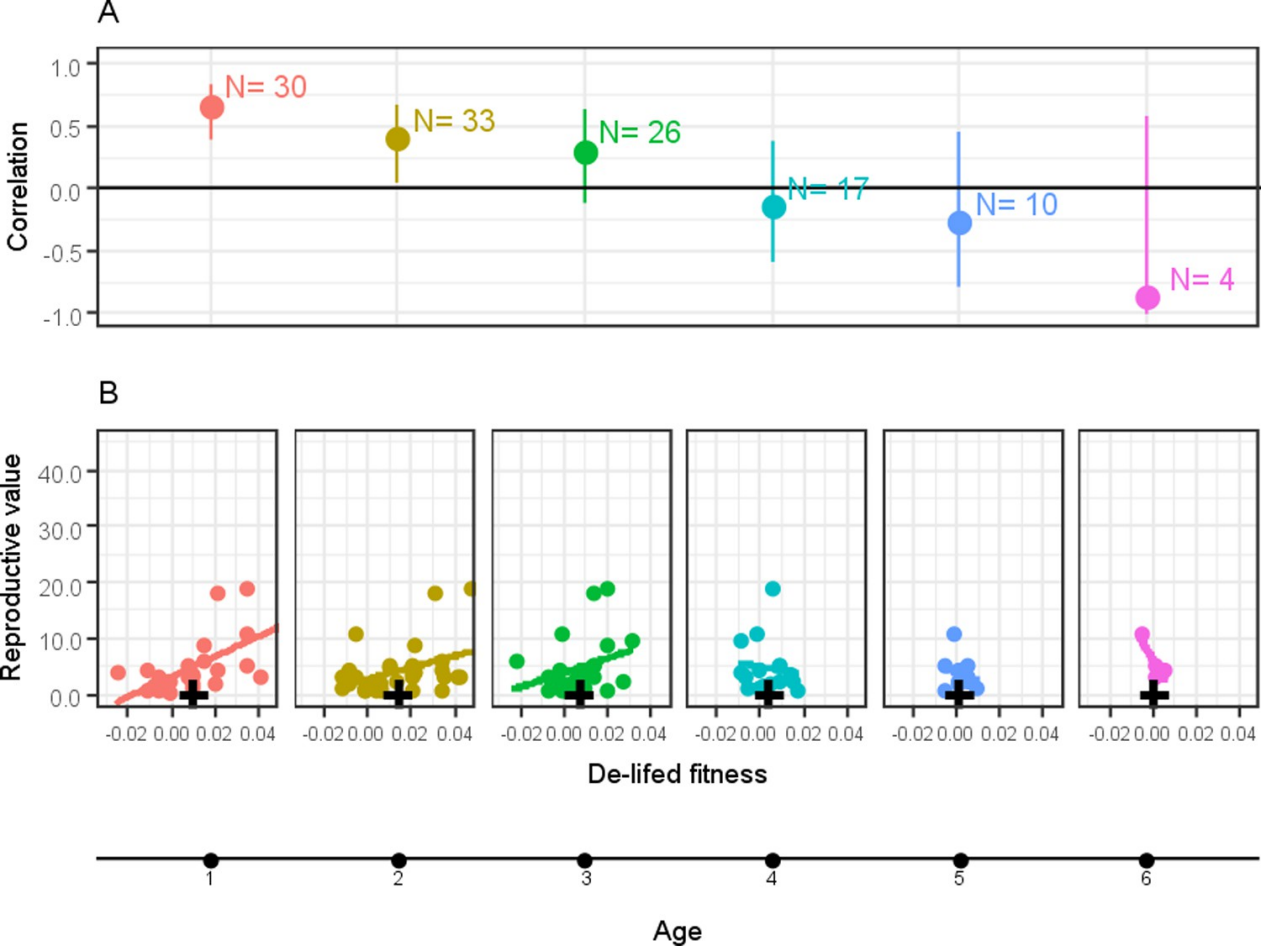
(A) Correlation between reproductive value and de-lifed fitness by age class (in years), with 95% confidence intervals. N represents the sample size. Older age classes have lower sample sizes because fewer individuals survive to that age. (B) Correlation between de-lifed fitness and reproductive value. The black cross represents mean de-lifed fitness for the respective age. Colours represent the same age class.

The fitness proxies all had positive or no association with the reproductive values (Fig 2). De-lifed fitness, IGR and LRS for recruits were all statistically significantly correlated with the reproductive values. There were no statistically significant differences between the IGR and LRS correlation coefficients, which also did not differ significantly from de-lifed fitness at recruit stage. None of the other correlation estimates were statistically significant (Table 1). De-lifed fitness at ages 1 and 2 significantly correlated with reproductive value, but in older age classes the correlation estimate was not statistically significant (Fig 3A and 3B).

**Table 1:** Mean and standard deviation for short-term fitness proxies at different offspring stages, and de-lifed fitness at different ages.

	Mean	SD
**LRS Eggs**	23.78	18.02
**LRS Broods**	5.80	4.26
**LRS Hatchlings**	20.30	16.04
**LRS Fledglings**	10.24	7.85
**LRS Recruits**	3.42	3.36
**De-lifing**	0.01	0.03
**IGR eggs**	4.24	1.53
**IGR hatchlings**	3.71	1.37
**IGR fledglings**	2.43	0.99
**IGR recruits**	1.13	0.78
**De-lifing–Age 1**	0.0003	0.0198
**De-lifing–Age 2**	0.0056	0.0170
**De-lifing–Age 3**	0.0027	0.0115
**De-lifing–Age 4**	0.0023	0.0087
**De-lifing–Age 5**	0.0033	0.0048
**De-lifing–Age 6**	0.0005	0.0046

There was a significant positive relationship between lineage survival odds and de-lifed fitness, LRS at recruitment and fledgling stages, and IGR at recruitment (Table 2). The estimated slopes for the de-lifed fitness and LRS recruits were significantly higher than the slopes of IGR and LRS at fledgling stage as their 95% confidence intervals are non-overlapping, indicating that fitness measured at the recruitment stage for these two metrics is a better predictor of lineage survival. There were no statistically significant differences between IGR and LRS at the same offspring stage (Table 2) and de-lifed fitness also was not significantly different from IGR and LRS at recruit stage.

**Table 2:** Results of binary logistic regressions with lineage survival as a response variable. l-95% CI and u-95% CI are the lower and upper boundaries of the 95% confidence interval for the slope, respectively.

Variable	Slope	l-95% CI	u-95% CI	p value
LRS eggs	0.42	-0.07	0.95	0.101
LRS hatchlings	0.45	-0.08	0.95	0.063
LRS fledglings	1.06	0.41	1.71	0.001*
LRS recruits	3.26	1.73	4.74	0.001*
LRS broods	0.47	-0.02	0.96	0.056
IGR eggs	0.24	-0.21	0.75	0.310
IGR hatchlings	0.35	-0.09	0.87	0.167
IGR fledglings	1.06	0.49	1.71	0.002
IGR recruits	2.65	1.67	3.70	0.001*
De-lifed fitness	3.01	1.74	4.32	0.001*

## Discussion

We showed that fitness proxies measured at recruit stage correlate best with long-term reproductive values and lineage survival, while fitness proxies measured at earlier stages are less accurate. Still, they are potentially useful in studies where recruits cannot be monitored.

Similar to a study by [4] lineage survival is low. While there was weak evidence for adifference in the rate of lineage survival between native and introduced lineages, the introduced lineages had significantly higher reproductive values. This indicates that the introduced lineages might have a fitness advantage over the native ones. For lineages that survived to 2018, there was wide variation in survival probability and reproductive value. The survival probability of a lineage is associated with its reproductive value in that year [4, 18]. While several lineages died out every year prior to 2007, all lineages that survived the bottleneck in 2008 also survived the next 10 years to 2018. Lineage extinctions are expected to become less likely over the generations, as all founders with non-zero reproductive values become genealogical ancestors of all individuals in the future population after only a few generations [19, 22]. After a founder becomes an ancestor of all individuals in the current population, its lineage can only go extinct if the entire population goes extinct. During the 2008 bottleneck, the population size decreased significantly, shortening the time it took for all founders of persisting lineages to become the common ancestors of the current population members.

There was variation in reproductive value, with most lineages ranging from 0 to 10 but some reaching contributions of over four times that much. There was also large variation over time as lineages fluctuated. Lineage stabilisation is also visible in the pattern of lineage fluctuation through time after 2007, as the ranking of lineages based on reproductive value remains similar with the correlation consistently over 0.90, while lineage rankings fluctuated more until 2007 when the correlations were also lower. The rapidly increasing correlation between reproductive value in the final year and each of the previous years also shows a pattern of stabilisation, as found in other studies too [4, 18, 35]. Stabilisation is reached after 12 years, with the correlation exceeding 0.95 afterwards. Despite stabilisation, reproductive values fluctuated through time. As we examined reproductive values that are absolute rather than relative to population size, any change in population size is also reflected in the sum of the reproductive values the year before. The change in reproductive value occurs one year previously, because the estimates are based on reproducing offspring, which are only recognised in the next year and form the basis of next year’s population.

The fitness proxies based on the number of recruits outperformed all other fitness proxies in predicting reproductive values and lineage survival. Recruits are likely to be the best measure because they are adult individuals that reproduced, while other proxies include the uncertainty of survival to adulthood before reproduction even occurs. Given that sparrow offspring experience high rates of mortality, with only 14% of laid eggs successfully surviving to recruitment, mortality will have a big impact on reproductive values from short-term metrics measured at early offspring stages. For species with lower offspring mortality the age at which offspring are counted towards fitness may have less influence on the predictive power of short-term fitness metrics. While recruits are clearly the best predictor of long-term fitness, they are the most difficult to measure in most study systems, as it is rarely possible to monitor all offspring until their first reproduction. This highlights the importance of long-term isolated island population studies [36], as only in such studies is it possible to accurately estimate the number of genetic recruits that an individual produced.

We found no differences in the performance of de-lifed fitness, IGR or LRS in predicting reproductive values or lineage survival. A previous study on Ural owls (*Strix uralensis*) and collared flycatchers (*Ficedula albicollis*) found that LRS performed significantly better than IGR at fledgling stage in predicting reproductive values, while they both performed similarly at recruitment [10]. In song sparrows (*Melospiza melodia*), LRS also performed better than IGR and for both metrics, the association was stronger when fitness was measured at older development stages [4]. The correlation between reproductive value and different fitness proxies at recruit stage in our study was of similar strength as discovered in previous studies [4, 10]. Our study therefore confirms the inaccuracy of short-term fitness metrics in predicting long-term fitness, and supports previous findings that short-term fitness should be measured at later offspring development stages Unlike previous studies, we found no difference between LRS and IGR in their correlation with reproductive value, which could result from deviating life history traits of the species used in the studies.

In this study, annual de-lifed fitness at ages 1 and 2 were correlated with the reproductive values, but not at later ages. The correlation at age 1 with reproductive value was similar to that for lifetime de-lifed fitness, indicating that reproductive success in the first adult year may be sufficient to provide a good prediction of long-term fitness. Hence, individual reproductive performance in the first year may be an important proxy for an individual’s fitness at least in short-lived species such as the house sparrow.

There is, however, considerable variation that is not explained by the fitness metrics. A strong correlation between a short-term fitness metric and the reproductive value measured two decades later, during which the population has been exposed to varying environmental conditions and population fluctuations, is unlikely. The strength of the correlation will also depend on the additive genetic variance and heritability of reproductive success [4]. In particular, in our population annual fitness is somewhat heritable [37], and there has been significant demographic stochasticity in our population for which LRS and IGR metrics tested here were not designed [38].

In conclusion, by using reproductive values as a measure of long-term individual fitness we have shown that recruits, rather than earlier offspring stages, best predict reproductive values. Additionally, annual fitness measured in the first reproductive season is an equally good predictor of fitness as lifetime fitness measures. We therefore suggest that future studies should measure short-term fitness at higher offspring ages to better capture long-term fitness.
